# Supplementary material for: Aesthetic appraisals of literary style and emotional intensity in narrative engagement are neurally dissociable
Source: Commun Biol. 2021 Dec 16;4:1401. doi: 10.1038/s42003-021-02926-0 (PMC8677754; doi:10.1038/s42003-021-02926-0)
Supplement: Supplementary file 2 — Supplementary Materials [file 42003_2021_2926_MOESM2_ESM.pdf]

Supplementary materials

Table of Contents

Supplementary Figure 1: Visualization of behavioural ratings of the two stories (in Dutch)......... 3

Supplementary Figure 2: Visualization of behavioural ratings of the two stories (in Dutch)......... 3

Supplementary Table 1: Segments rated highest for literariness 4

Supplementary Figure 3: Correlations of percent signal changes in literariness-related regions of interest with one another and behavioural measures 6

Supplementary Table 2: Correlations of percent signal changes in literariness-related regions of interest with one another 7

Supplementary Table 3: Correlations of percent signal changes in literariness-related regions of interest with appreciation measures 8

Supplementary Table 4: Correlations of percent signal changes in literariness-related regions of interest with experiential measures 9

Supplementary Table 5: Correlations of percent signal changes in literariness-related regions of interest with individual differences in reading behaviour 10

Supplementary Table 6: Correlations of percent signal changes in literariness-related regions of interest with individual differences in social cognition.. 11

Supplementary Figure 4: Correlations of percent signal changes in emotion-related regions of interest with one another and behavioural measures 12

Supplementary Table 7: Correlations of percent signal changes in emotion-related regions of interest with one another 13

Supplementary Table 8: Correlations of percent signal changes in emotion-related regions of interest with appreciation measures 13

Supplementary Table 9: Correlations of percent signal changes in emotion-related regions of interest with experiential measures 14

Supplementary Table 10: Correlations of percent signal changes in emotion-related regions of interest with individual differences in reading behaviour 14

Supplementary Table 11: Correlations of percent signal changes in emotion-related regions of interest with individual differences in social cognition.. 16

Supplementary Method: Whole brain analysis with a smaller cluster-extent threshold 17

Supplementary Table12: Whole brain analysis with a smaller cluster-extent threshold 18

Supplementary Figure 5: Brain correlates of appraised literariness during narrative engagement 19

Supplementary Figure 6: Brain correlates of appraised emotional intensity during narrative engagement 19

Supplementary Discussion 20
Literariness 20
Emotional Intensity 20
General Discussion 21
Supplementary Table 13: Comparison of behavioural measures between fMRI and rater groups 22
Supplementary Note: English translations of the literary stories 24
De Mexicaanse Hond (The Mexican Dog) 24
De Muur (The Wall) 27
Supplementary Figure 7: Correlations among behavioural measures in fMRI group 30
Spearman's rho heatmap 30
Supplementary References 31

Supplementary Figure 1: Visualization of behavioural ratings of the two stories (in Dutch)

De Mexicaanse Hond (The Mexican Dog)

https://osf.io/rab2p/

Supplementary Figure 1: Visualization of the emotional intensity (font size) and literariness (color scale) ratings from the story De Mexicaanse Hond (English translation of the story in Supplementary Material 7).

Supplementary Figure 2: Visualization of behavioural ratings of the two stories (in Dutch)

De Muur (The Wall)

https://osf.io/4aqtu/

Supplementary Figure 2: Visualization of emotional intensity (font size) and literariness (color scale) ratings from the story De Muur (English translation of the story in Supplementary Material 7).

Supplementary Table 1: Segments rated highest for literariness

**Dutch Original**	**English Translation**
De Mexicaanse Hond	The Mexican Dog
Meneer Kuisters van de viswinkel, waar ik af en toe vrijdagsmiddags na school gesneden zalm voor mijn moeder moest halen, was een lange, **schonkige** man **wiens gezicht hoofdzakelijk uit plooien bestond.**	Mr. Kuisters from the fish shop, where I occasionally had to pick up sliced salmon for my mom on Friday afternoons after school, was a tall, **bony** man **whose face consisted mainly of wrinkles.**
Tonia zat aan tafel naar haar handen te kijken die voor haar op het **pluche tafelkleed** lagen; een **bleek kind met lichte, waterige ogen en vlasachtig haar.**	Tonia sat at the table staring at her hands lying in front of her on the **plush tablecloth**; a **pale child with light, watery eyes and hair like flax.**
Haar mond hing half open, **haar witte gezicht glom alsof het met vet was ingesmeerd.**	Her mouth hung half open; **her white face gleamed like it was smeared with grease.**
De ruimte werd nog meer verkleind door een enorme lamp met **franje die als een parasol** boven onze hoofden hing.	The room was further cramped by a huge lamp with **tassels that hung like a parasol** over our heads.
Ik zag de **rode adertjes in zijn vochtige ogen** en hoe **zijn pupillen heen en weer schoten** — hij wilde zien wat ik hoorde.	I saw the **red veins in his watery eyes** and how **his pupils darted back and forth** - he wanted to see what I was hearing.
Hier is de HDO, de Hilversumse Draadloze Omroep,' riep iemand en na een paar onverstaanbare zinnen sloeg er keiharde muziek tegen mijn trommelvliezen, alsof mijn hoofd **bekneld zat in de hoorn van mijn vaders pathefoon.**	"Here's HDO, the Hilversum Radio Broadcasting," someone shouted, and after a few unintelligible sentences some deafening music hit against my eardrums, as if my head **was jammed into the horn of my father's gramophone.**
Na een tijdje verloste meneer Kuisters me **onzacht** van de koptelefoon.	After a while, Mr. Kuisters **ungently** freed of the headphones.
Met **suizende oren** en **bonzende slapen** stond ik op en liep naar de deur.	With **ringing ears** and **throbbing temples** I stood up and ran to the door.
Het bleef een **barse** monoloog.	It remained a **grim** monologue.
Ik verstond alleen het woord 'Juden', dat de man steeds vaker uitsprak, en op een steeds **verachtelijker** toon, **alsof hij ertegen trapte.**	I only understood the word "Juden*", which the man uttered with increasing frequency, in an increasingly **contemptuous** tone, **as if he was kicking against it.** **Juden=Jews in German*
Hij overstemde zelfs de **roffelende wastafelkraan** die ik open had gezet omdat ik benieuwd was of ik hem dan nog zou kunnen horen.	He even drowned out (the sound of) the **rumbling sink faucet** that I had set open because I was curious whether I could still hear him (against the running water).
De Muur	The Wall
Met mijn wijsvinger **scharrel** ik **behoedzaam** het laatste restje uit een blik.	With my index finger I **carefully scratch** the leftovers from the can.
Ik trek mijn rok op, zak op handen en knieën en **sleur mijn** oude **lijf** naar de woonkamer.	I lift up my skirt, drop to my hands and knees and **drag my** old **body** to the living room.
De man gedraagt zich als een **roofdier.**	The man behaves like a **predatory beast.**
**De muur zwijgt.**	**The wall keeps silent.**
Geen fout meer maken, **zijn opmerkzaamheid niet** naar de **muur richten**, muisstil zijn.	Don’t make any more mistakes, **do not direct his attention** to the **wall**, be quiet as a mouse.
De buren hebben zich **teruggetrokken in het cocon van de slaap.**	The neighbours have **withdrawn into the cocoon of sleep.**
Zijn er al buren wakker of zwerven ze nog **in de geluidloze wereld van de slaap**.	Are there neighbours awake yet, or do they still wander **in the soundless world of sleep.**

**Vanaf welk uur is de nacht geen nacht meer?**	**From which hour onwards will night no longer be night?**
Misschien heeft de man gewacht tot de **vermoeidheid me verdooft.**	Perhaps the man waited until **fatigue numbed me**.
Waarom schijnt er nog steeds geen licht door het gordijn, **waarop wacht het ochtendlicht?**	Why is there still no daylight shining through the curtains yet, **what is the morning light waiting for?**
De **ketens van de slaap** kunnen me niet meer verontrusten.	The **chains of sleep** shan't disturb me no more.
Ik **takel** mijn lijf op, strompel naar de keuken en open een blik.	I **hoist** up my body, stumble to the kitchen and open a can.
Mijn blik glijdt naar de **tochtspleet,** de onbedekte strook tussen de onderkant van de deur en de drempel.	My gaze glides to the **'tochtspleet*'**, the bare strip between the bottom edge of the door and the floor. **tochtspleet (literally 'draft slit') is the word referring to the drafty gap underneath doors; tocht is also an old word for 'thought', so it could also be used as 'thought gap'*
**Mijn scherpe vingernagels schrammen mijn knieën.**	**My sharp-edged fingernails gouge my knees.**

Supplementary Table 1: All words with mean literariness ≥1.75 (i.e., more than 75% raters rated as literary) from the two stories are in bold.

Supplementary Figure 3: Correlations of percent signal changes in literariness-related regions of interest with one another and behavioural measures

Spearman's rho heatmap

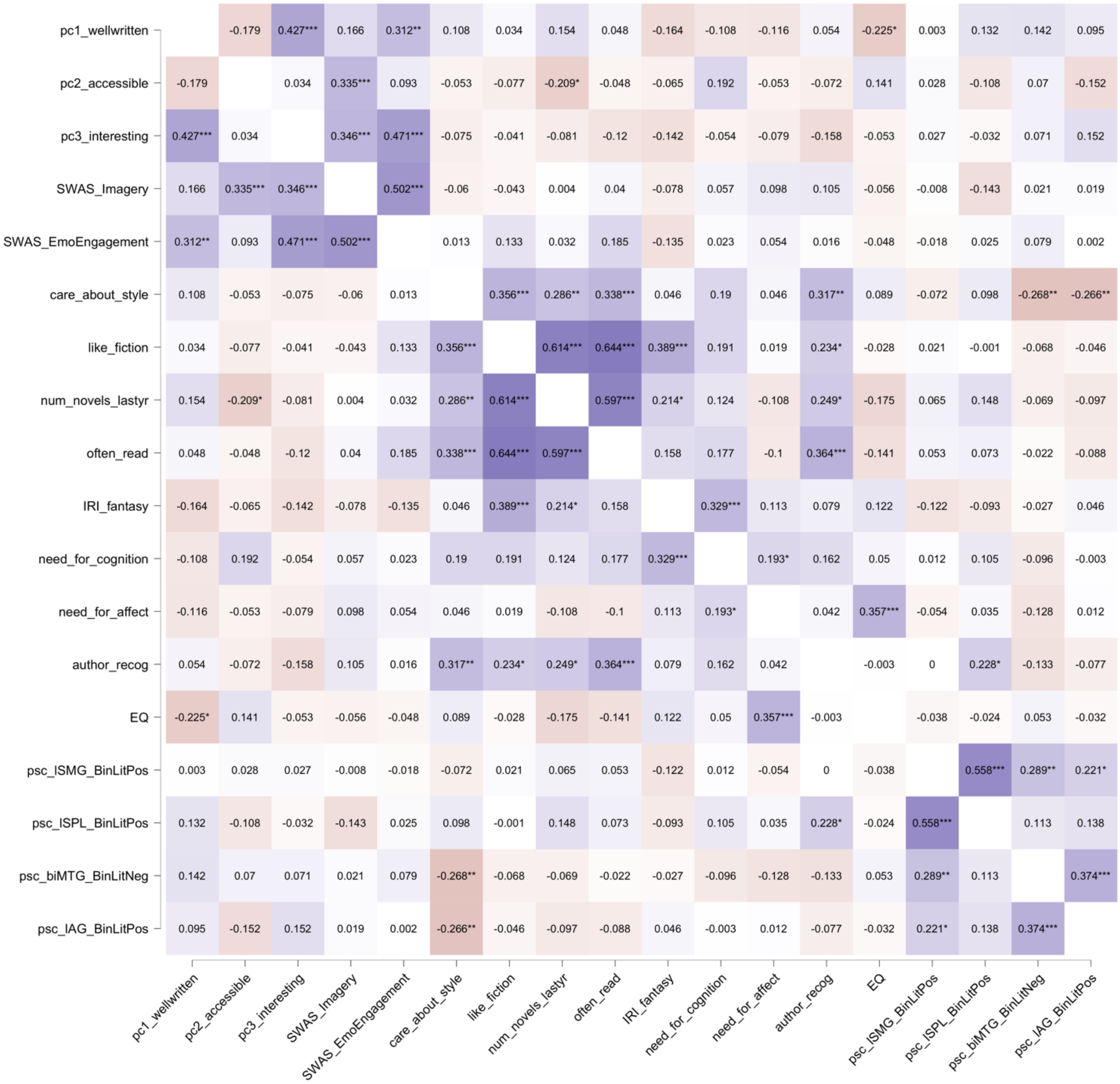

Supplementary Figure 3: Correlation heatmap of percent signal changes in literariness ROIs with story appreciation and transportation measures, individual differences in reading preferences, reward, and social cognition measures.

Supplementary Table 2: Correlations of percent signal changes in literariness-related regions of interest with one another

Spearman's Correlations of PSC in literariness ROIs

			Spearman's rho		p
psc_lSMG_BinLitPos	-	psc_lSPL_BinLitPos	0.558	***	< .001
psc_lSMG_BinLitPos	-	psc_lAG_BinLitPos	0.221	*	0.024
psc_lSMG_BinLitPos	-	psc_biMTG_BinLitNeg	0.289	**	0.003
psc_lSPL_BinLitPos	-	psc_lAG_BinLitPos	0.138		0.162
psc_lSPL_BinLitPos	-	psc_biMTG_BinLitNeg	0.113		0.252
psc_lAG_BinLitPos	-	psc_biMTG_BinLitNeg	0.374	***	< .001

* p < .05, ** p < .01, *** p < .001

Supplementary Table 2: Correlation of percent signal changes in literariness ROIs.

Supplementary Table 3: Correlations of percent signal changes in literariness-related regions of interest with appreciation measures

Spearman's Correlations with aesthetic evaluation measures

			Spearman's rho	p
pc1_wellwritten	-	psc_lSMG_BinLitPos	0.003	0.974
pc1_wellwritten	-	psc_lSPL_BinLitPos	0.132	0.184
pc1_wellwritten	-	psc_lAG_BinLitPos	0.095	0.339
pc1_wellwritten	-	psc_biMTG_BinLitNeg	0.142	0.154
pc2_accessible	-	psc_lSMG_BinLitPos	0.028	0.777
pc2_accessible	-	psc_lSPL_BinLitPos	-0.108	0.276
pc2_accessible	-	psc_lAG_BinLitPos	-0.152	0.125
pc2_accessible	-	psc_biMTG_BinLitNeg	0.070	0.480
pc3_interesting	-	psc_lSMG_BinLitPos	0.027	0.790
pc3_interesting	-	psc_lSPL_BinLitPos	-0.032	0.749
pc3_interesting	-	psc_lAG_BinLitPos	0.152	0.125
pc3_interesting	-	psc_biMTG_BinLitNeg	0.071	0.475

* $p < .05$, ** $p < .01$, *** $p < .001$

Supplementary Table 3: Correlation of percent signal changes in literariness ROIs with story-specific aesthetic measures. Pc1, 2, and 3 are principal components of the 10-adjective story appreciation measures.

Supplementary Table 4: Correlations of percent signal changes in literariness-related regions of interest with experiential measures

Spearman's Correlations with experiential measures

			Spearman's rho	p
SWAS_Imagery	-	psc_lSMG_BinLitPos	-0.008	0.934
SWAS_Imagery	-	psc_lSPL_BinLitPos	-0.143	0.151
SWAS_Imagery	-	psc_lAG_BinLitPos	0.019	0.851
SWAS_Imagery	-	psc_biMTG_BinLitNeg	0.021	0.835
SWAS_EmoEngagement	-	psc_lSMG_BinLitPos	-0.018	0.854
SWAS_EmoEngagement	-	psc_lSPL_BinLitPos	0.025	0.803
SWAS_EmoEngagement	-	psc_lAG_BinLitPos	0.002	0.983
SWAS_EmoEngagement	-	psc_biMTG_BinLitNeg	0.079	0.430

* $p < .05$, ** $p < .01$, *** $p < .001$

Supplementary Table 4: Correlations of percent signal changes in literariness ROIs with story-specific experiential measures.

Supplementary Table 5: Correlations of percent signal changes in literariness-related regions of interest with individual differences in reading behaviour

Spearman's Correlations with reading behavior

			Spearman's rho		p
care_about_style	-	psc_lSMG_BinLitPos	-0.072		0.470
care_about_style	-	psc_lSPL_BinLitPos	0.098		0.323
care_about_style	-	psc_lAG_BinLitPos	-0.266	**	0.006
care_about_style	-	psc_biMTG_BinLitNeg	-0.268	**	0.006
like_fiction	-	psc_lSMG_BinLitPos	0.021		0.832
like_fiction	-	psc_lSPL_BinLitPos	-5.802e -4		0.995
like_fiction	-	psc_lAG_BinLitPos	-0.046		0.646
like_fiction	-	psc_biMTG_BinLitNeg	-0.068		0.493
num_novels_lastyr	-	psc_lSMG_BinLitPos	0.065		0.512
num_novels_lastyr	-	psc_lSPL_BinLitPos	0.148		0.134
num_novels_lastyr	-	psc_lAG_BinLitPos	-0.097		0.329
num_novels_lastyr	-	psc_biMTG_BinLitNeg	-0.069		0.484
often_read	-	psc_lSMG_BinLitPos	0.053		0.591
often_read	-	psc_lSPL_BinLitPos	0.073		0.464
often_read	-	psc_lAG_BinLitPos	-0.088		0.376
often_read	-	psc_biMTG_BinLitNeg	-0.022		0.828
author_recog	-	psc_lSMG_BinLitPos	-2.892e -4		0.998
author_recog	-	psc_lSPL_BinLitPos	0.228	*	0.020
author_recog	-	psc_lAG_BinLitPos	-0.077		0.437
author_recog	-	psc_biMTG_BinLitNeg	-0.133		0.177

* p < .05, ** p < .01, *** p < .001

Supplementary Table 5: Correlation of percent signal changes in literariness ROIs with individual reading behavior.

Supplementary Table 6: Correlations of percent signal changes in literariness-related regions of interest with individual differences in social cognition

Spearman's Correlations with reward and social cognition measures

			Spearman's rho	p
IRI_fantasy	-	psc_lSMG_BinLitPos	-0.122	0.217
IRI_fantasy	-	psc_lSPL_BinLitPos	-0.093	0.348
IRI_fantasy	-	psc_lAG_BinLitPos	0.046	0.645
IRI_fantasy	-	psc_biMTG_BinLitNeg	-0.027	0.783
need_for_cognition	-	psc_lSMG_BinLitPos	0.012	0.900
need_for_cognition	-	psc_lSPL_BinLitPos	0.105	0.288
need_for_cognition	-	psc_lAG_BinLitPos	-0.003	0.975
need_for_cognition	-	psc_biMTG_BinLitNeg	-0.096	0.335
need_for_affect	-	psc_lSMG_BinLitPos	-0.054	0.583
need_for_affect	-	psc_lSPL_BinLitPos	0.035	0.727
need_for_affect	-	psc_lAG_BinLitPos	0.012	0.901
need_for_affect	-	psc_biMTG_BinLitNeg	-0.128	0.195
EQ	-	psc_lSMG_BinLitPos	-0.038	0.703
EQ	-	psc_lSPL_BinLitPos	-0.024	0.812
EQ	-	psc_lAG_BinLitPos	-0.032	0.748
EQ	-	psc_biMTG_BinLitNeg	0.053	0.590

* p < .05, ** p < .01, *** p < .001

Supplementary Table 6: Correlation of percent signal changes in literariness ROIs with individual reward and social cognition measures.

Supplementary Figure 4: Correlations of percent signal changes in emotion-related regions of interest with one another and behavioural measures

Spearman's rho heatmap

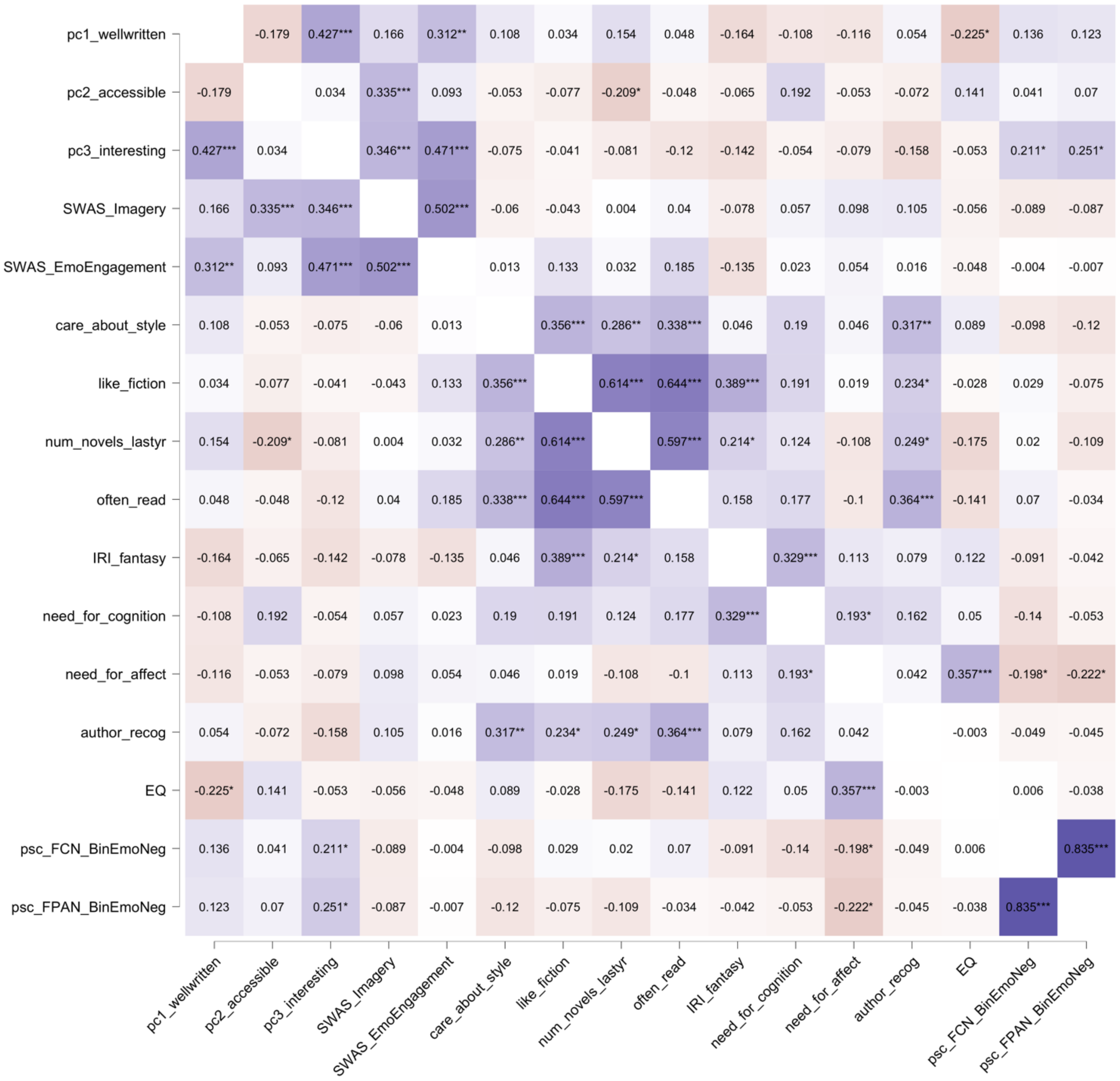

Supplementary Figure 4: Correlation heatmap of percent signal changes in emotional intensity ROIs with story appreciation and transportation measures, individual differences in reading preferences, reward, and social cognition measures.

Supplementary Table 7: Correlations of percent signal changes in emotion-related regions of interest with one another

Spearman's Correlations of PSC in emotional intensity ROIs

			Spearman's rho		p
psc_FCN_BinEmoNeg	-	psc_FPAN_BinEmoNeg	0.835	***	< .001

* p < .05, ** p < .01, *** p < .001

Supplementary Table 7: Correlation of percent signal changes in emotional intensity ROIs.

Supplementary Table 8: Correlations of percent signal changes in emotion-related regions of interest with appreciation measures

Spearman's Correlations with aesthetic evaluation measures

			Spearman's rho		p
pc1_wellwritten	-	psc_FCN_BinEmoNeg	0.136		0.170
pc1_wellwritten	-	psc_FPAN_BinEmoNeg	0.123		0.217
pc2_accessible	-	psc_FCN_BinEmoNeg	0.041		0.679
pc2_accessible	-	psc_FPAN_BinEmoNeg	0.070		0.483
pc3_interesting	-	psc_FCN_BinEmoNeg	0.211	*	0.032
pc3_interesting	-	psc_FPAN_BinEmoNeg	0.251	*	0.011

* p < .05, ** p < .01, *** p < .001

Supplementary Table 8: Correlation of percent signal changes in emotional intensity ROIs with story-specific aesthetic measures. Pc1, 2, and 3 are principal components of the 10-adjective story appreciation measures.

Supplementary Table 9: Correlations of percent signal changes in emotion-related regions of interest with experiential measures

Spearman's Correlations with experiential measures

			Spearman's rho	p
SWAS_Imagery	-	psc_FCN_BinEmoNeg	-0.089	0.375
SWAS_Imagery	-	psc_FPAN_BinEmoNeg	-0.087	0.382
SWAS_EmoEngagement	-	psc_FCN_BinEmoNeg	-0.004	0.966
SWAS_EmoEngagement	-	psc_FPAN_BinEmoNeg	-0.007	0.947

* p < .05, ** p < .01, *** p < .001

Supplementary Table 9: Correlations of percent signal changes in emotional intensity ROIs with story-specific experiential measures.

Supplementary Table 10: Correlations of percent signal changes in emotion-related regions of interest with individual differences in reading behaviour

Spearman's Correlations with reading behavior

			Spearman's rho	p
care_about_style	-	psc_FCN_BinEmoNeg	-0.098	0.323
care_about_style	-	psc_FPAN_BinEmoNeg	-0.120	0.225
like_fiction	-	psc_FCN_BinEmoNeg	0.029	0.769
like_fiction	-	psc_FPAN_BinEmoNeg	-0.075	0.452
num_novels_lastyr	-	psc_FCN_BinEmoNeg	0.020	0.841
num_novels_lastyr	-	psc_FPAN_BinEmoNeg	-0.109	0.270
often_read	-	psc_FCN_BinEmoNeg	0.070	0.482
often_read	-	psc_FPAN_BinEmoNeg	-0.034	0.731
author_recog	-	psc_FCN_BinEmoNeg	-0.049	0.618
author_recog	-	psc_FPAN_BinEmoNeg	-0.045	0.650

* $p < .05$, ** $p < .01$, *** $p < .001$

Supplementary Table 10: Correlation of percent signal changes in emotional intensity ROIs with individual reading behavior.

Supplementary Table 11: Correlations of percent signal changes in emotion-related regions of interest with individual differences in social cognition

Spearman's Correlations with reward and social cognition measures

			Spearman's rho		p
IRI_fantasy	-	psc_FCN_BinEmoNeg	-0.091		0.356
IRI_fantasy	-	psc_FPAN_BinEmoNeg	-0.042		0.672
need_for_cognition	-	psc_FCN_BinEmoNeg	-0.140		0.157
need_for_cognition	-	psc_FPAN_BinEmoNeg	-0.053		0.596
need_for_affect	-	psc_FCN_BinEmoNeg	-0.198	*	0.044
need_for_affect	-	psc_FPAN_BinEmoNeg	-0.222	*	0.023
EQ	-	psc_FCN_BinEmoNeg	0.006		0.948
EQ	-	psc_FPAN_BinEmoNeg	-0.038		0.704

* $p < .05$, ** $p < .01$, *** $p < .001$

Supplementary Table 11: Correlation of percent signal changes in emotional intensity ROIs with individual reward and social cognition measures.

Supplementary Method: Whole brain analysis with a smaller cluster-extent threshold

We additionally implemented a group-level analysis with a smaller cluster-extent threshold based on Monte Carlo simulation of Gaussian fields[1,2] using Matlab script cluster_threshold_beta.m (https://sites.google.com/bc.edu/sd-slotnick/publications/scripts-and-stimuli, retrieved on Oct 12, 2019). Smoothness of residual image was estimated by spm_est_smoothness.m in SPM12 (FWHM(x,y,z)=(6,8,6)) and a cluster threshold of 49 voxels was obtained using voxel-wise $p<0.001$, cluster-wise corrected $p<0.05$ at 8 mm FWHM and 2,500 simulations. These findings are very similar to Lehne and colleagues' (2015)[3] findings linked to suspense in narratives.

Supplementary Table12: Whole brain analysis with a smaller cluster-extent threshold

Contrast		Region	Cluster Size	x	y	z	Max. t-value (df=51)
Emo+	L	Medial Frontal Gyrus	51	-4	56	26	3.8635
	L	Superior Temporal Gyrus, Angular Gyrus, Supramarginal Gyrus	89	-60	-58	26	3.9642
	L	Superior Frontal Gyrus	144	-6	24	62	4.4389
Emo-	L	Cerebellum Posterior Lobe	466*	-42	-54	-32	4.9969
	R	Parahippocampal Gyrus, Fusiform Gyrus	312*	30	-30	-20	5.104
	L	Parahippocampal Gyrus, Fusiform Gyrus	102	-30	-28	-20	4.7534
	R	Middle + Inferior Temporal Gyrus	197	56	-52	-12	6.8882
	L	Lingual Gyrus	69	-12	-78	-10	3.7247
	L	Sub-Gyral, Middle Temporal Gyrus	96	-48	-50	-6	4.7572
	-	R Inferior Parietal Lobule, L+R Mid and Posterior Cingulate Gyri, L+R Postcentral Gyri, L+R Precentral Gyri, L+R Precunei, L+R Cunei, R Middle Frontal Gyrus, L+R Medial Frontal Gyri	13793*	46	-40	52	8.5226
	R	Middle + Inferior Frontal Gyrus (pars triangularis, pars orbitalis)	1769*	46	38	18	6.3787
	R	Medial Frontal Gyrus	159	12	50	-10	4.5507
	R	Insula, Inferior Frontal Gyrus	140	36	20	2	4.337
	L	Insula	116	-40	-6	2	4.4448
	R	Insula, Rolandic operculum, Precentral Gyrus, Superior Temporal Gyrus, Heschl's Gyrus	263	42	-16	10	4.3524
	R	Sub-Gyral, Calcarine Sulcus	152	24	-54	14	4.0172
	L	Middle Frontal Gyrus	51	-40	46	12	3.9004
	R	Inferior Frontal Gyrus (pars opercularis), Precentral Gyrus	246	46	6	28	4.3785
	L	Middle Frontal Gyrus, Inferior Frontal Gyrus (pars triangularis)	91	-40	32	24	4.1614
	L	Inferior Frontal Gyrus (pars opercularis), Precentral Gyrus	76	-42	4	28	3.9561
Lit+	L	Inferior Parietal Lobule, Supramarginal Gyrus	571*	-52	-44	40	4.7988
	L	Precuneus, Superior Parietal Lobule	570*	-10	-66	54	5.2963
	L	Inferior Frontal Gyrus (pars triangularis), Middle Frontal Gyrus	136	-44	32	20	4.132
Lit-	L	Superior + Middle Temporal Gyrus, Heschl's Gyrus	986*	-46	-28	2	6.1302
	R	Superior + Middle Temporal Gyrus, Heschl's Gyrus	1146*	52	-18	4	5.9044
	-	R+L Precunei	177	4	-56	26	3.9658

Supplementary Table 12: fMRI group-level results using GLM modeling semantic events with Gaussian field simulation-based correction (cluster-wise corrected $p<0.05$, voxel-wise corrected $p<0.001$). "Emo" or "Lit" indicates correlation with either emotional arousal or literariness regressor. "+" indicates a positive correlation with the regressor and "-" indicates a negative correlation. "*" indicates clusters that also passed permutation correction using SnPM (cluster-wise corrected $p<0.05$, cluster-forming threshold $p<0.001$). The location of the peak t-value is always reported as the first anatomical region in the cluster.

Supplementary Figure 5: Brain correlates of appraised literariness during narrative engagement

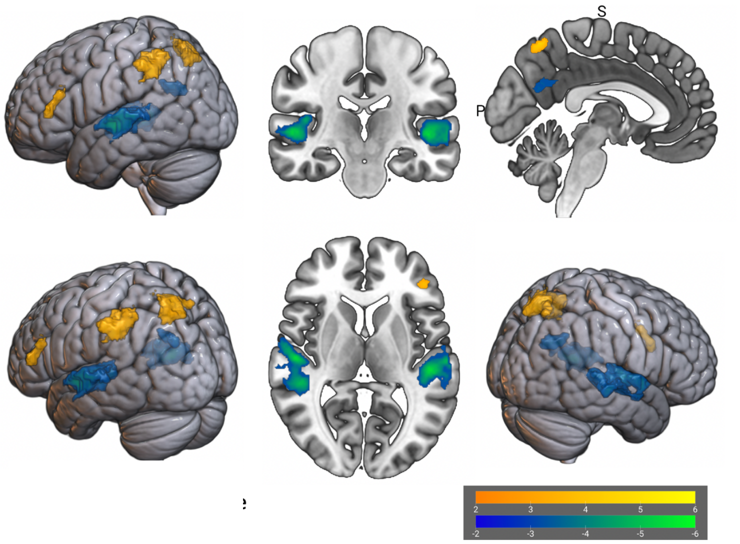

Supplementary Figure 5: Positive brain correlates (in yellow) and negative correlates (in blue) of appraised literariness during narrative engagement significant at Gaussian field simulation-based cluster-extent threshold, which include smaller clusters not present with permutation-based thresholding. Cluster-wise corrected $p<0.05$, voxel-wise corrected $p<0.001$.

Supplementary Figure 6: Brain correlates of appraised emotional intensity during narrative engagement

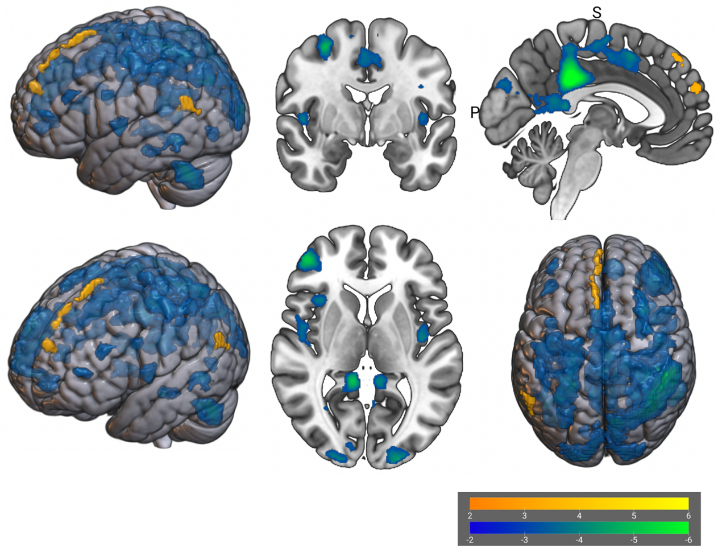

Supplementary Figure 6: Positive brain correlates (in yellow) and negative correlates (in blue) of appraised emotional intensity during narrative engagement significant at Gaussian field simulation-based cluster-extent threshold, which include smaller clusters not present with permutation-based thresholding. Cluster-wise corrected p<0.05, voxel-wise corrected p<0.001.

Supplementary Discussion

Literariness

Bilateral IFG, especially left IFG, are core regions for language processing and have been shown to be involved in emotional processing of negatively-valenced stories[4] and suspense in narratives[3]. Our results (Supplementary Figure 5) found activation in left IFG and left inferior frontal sulcus bordering on left middle frontal gyrus. The correlation results (see main discussion) seem to suggest language-related function in the left IFG ROI defined using Neurosynth, which is slightly more inferior to the activation cluster. It remains an open question if there is a functional gradient along the cranial-caudal axis of left IFG, similar to that along the ventral-dorsal axis[5].

Emotional Intensity

In previous studies, left SFG was found to be involved in mediating attention to valenced words[6], causal attribution[7,8], and contextualized language comprehension[9,10]. We suspect that our emotional intensity ratings may be similar to suspense ratings since both stories used in this study are suspenseful with no positive relieves. Interestingly, Wegrzyn and colleagues' (2017)[6] study showed this region in the left SFG to be co-activated with left IFG during higher-level interaction between emotion (valenced words) and auditory attention, which our results did not find (Supplementary Figure 6).

The left supramarginal gyrus at the anterior temporoparietal junction (TPJ) and the right superior temporal sulcus are crucial components of the ToM/mentalizing network[11–14] and predictive inference[15]. It

is not surprising that increased activity in these regions was positively correlated with judgments of emotional intensity and suspense indicated that suspenseful and emotionally intense parts of a narrative are linked to mentalizing. These sections might increase participants' probability to rely on imagining story characters' thoughts and feelings. It is also possible that the feeling of suspense is a result of increasing cognitive appraisal of the complex social and emotional situation surrounding the story characters based on appraisal from the perspective of a privileged witness[16]. Granted, this group-level result does not speak to individual strategies and individuals may have a preferred way of engaging with literature either through mentalizing, empathizing, or both[17].

General Discussion

Future research should try to disentangle different strategies in emotional involvement. With a similar experimental design as Wallentin et al. (2011)[18], we did not find increased activation to highly emotional content in the thalamus, amygdala, temporal cortices or motor regions. Wallentin and colleagues[18] used 3 different types of predictors: a bivariate emotion predictor (1 for positive emotion and -1 for negative emotion), a parametric emotional valence predictor (no significant activations reported), and a parametric emotional intensity predictor similar to ours. In our study the results for emotion may be targeted at appraisal of suspense. One important difference between the two studies is that Wallentin and colleagues used a well-known childhood story, *The Ugly Duckling* by H.C. Anderson, while we used literary stories that were novel to participants. Familiarity with the story may be linked with autobiographical memory retrieval during the more memorable, emotional segments, which may explain the strong activation in the temporal cortices seen in Wallentin et al.[19,20]. Future research is needed to better understand the differences between processing familiar and unfamiliar narratives.

Supplementary Table 13: Comparison of behavioural measures between fMRI and rater groups

Questionnaire Name (story-specific)	Question (on a scale of 1-4 with 1 being "completely disagree" and 4 being "completely agree")	De Mexicaanse Hond				t-test (Bonferroni)		De Muur				t-test (Bonferroni)	
		fMRI Group		Rater Group		t	p	fMRI Group		Rater Group		t	p
		Mean	SD	Mean	SD			Mean	SD	Mean	SD		
Appreciation Questionnaire	interesting	2.04	0.94	2.63	0.98	-3.16	0.10	1.73	0.95	3.04	0.85	-7.77	1.5E-9
	well-written	2.06	0.93	3.00	0.80	-5.56	1.1E-5	1.73	0.77	3.20	0.66	-10.6	2.4E-16
	of high literary quality	2.24	0.99	2.94	0.79	-4.04	5.1E-3	2.21	0.89	3.04	0.78	-5.80	8.6E-5
	easy to understand	2.29	0.92	3.33	0.87	-5.93	2.0E-6	1.88	1.02	3.26	0.81	-7.98	6.4E-10
	accessible	2.41	0.92	3.17	0.88	-4.28	2.0E-3	2.27	1.01	3.07	0.77	-5.17	6.5E-4
	thrilling	2.33	1.01	2.80	1.05	-2.30	1.1	1.96	0.97	3.15	0.98	-6.04	4.0E-7
	beautiful	2.75	0.84	2.31	0.99	2.40	0.87	2.35	0.93	2.59	0.88	-0.32	7.9
	fascinating	2.16	0.92	2.67	1.05	-2.65	0.45	2.04	1.01	2.85	0.90	-4.87	1.4E-3
	emotional	2.78	0.94	*	*	-	-	2.37	1.03	*	*	-	-
	sad	2.96	0.92	*	*	-	-	3.29	0.91	*	*	-	-
SWAS (Imagery)	Sometimes I had the feeling that I could see through the eyes of the main character.	1.96	0.88	3.00	0.89	-6.02	1.3E-6	1.96	0.99	3.17	0.80	-8.50	2.5E-8
	While reading I saw situations that were described to me as if I was there myself.	1.75	0.68	3.02	0.94	-7.96	1.6E-10	1.75	0.81	3.15	0.96	-9.34	5.8E-11
	Sometimes I had the feeling that I was in the environment in which the story took place.	2.12	0.92	2.85	1.04	-3.87	9.1E-3	2.04	0.99	3.02	0.90	-6.11	2.9E-5
	Sometimes I saw the environment in which the story takes place in front of me.	1.75	0.65	3.28	0.90	-10.0	5.5E-15	1.52	0.70	3.39	0.76	-14.2	3.6E-22
	While reading, I saw situations that were described to me as if I were a silent spectator.	1.98	0.87	2.74	1.01	-4.14	3.4E-3	1.94	0.87	2.87	1.13	-5.16	3.5E-4
	While reading this story I saw a picture of the main character in front of me.	2.67	1.17	2.30	1.00	1.78	3.7	2.19	1.05	2.72	1.07	-1.58	0.55
SWAS (Emotional	I felt like the main character felt.	2.79	1.04	2.37	1.00	2.12	1.8	2.96	1.05	2.17	0.97	4.26	4.6E-3

Engagement)	I could empathize with the characters.	2.21	0.96	2.76	0.93	-2.99	0.17	2.29	1.02	2.54	1.02	-1.70	10
	I sympathized with the main character.	2.13	0.89	2.63	0.98	-2.73	0.35	2.10	0.93	2.96	0.89	-5.64	1.8E-4
	I shared the protagonist's emotions.	2.63	0.93	2.43	1.00	1.11	13	2.87	0.89	2.52	1.06	1.36	3.4
	I felt sorry for the protagonist.	2.48	1.04	2.48	1.06	-0.003	48	2.13	1.10	3.06	0.94	-4.50	5.5E-4
	As I read, I could imagine what it would feel like to be in the protagonist's shoes.	2.27	0.95	2.85	1.07	-2.96	0.18	2.40	1.09	2.70	1.04	-2.12	7.2
	I knew exactly which emotions the characters experienced.	2.77	0.94	2.41	1.00	1.92	2.8	2.77	0.96	2.46	0.97	1.90	5.0
	I could imagine how the main character felt.	2.08	0.84	2.85	0.98	-4.39	1.3E-3	2.12	0.92	2.94	0.94	-5.51	6.1E-4
	I understood how the main character felt.	2.33	0.90	2.80	0.98	-2.57	0.56	2.31	0.98	2.67	1.08	-2.01	3.7

		fMRI Group		Rater Group		t	p
		Mean	SD	Mean	SD		
Reading Habits (person-specific)	How much do you like fiction? (1-7 Likert scale with 7 being "I love fiction")	5.19	1.48	5.28	1.58	-0.29	37
	How many novels did you read last year?	7.64	12.01	6.52	6.45	0.60	27

Supplementary Table 13: Behavioural measures of fMRI and rater groups assessing their overall engagement with DH and DM and individual reading habits. The rater group originally answered these questions on a scale of 0-100, and the results were converted to be on a scale of 1-4 (for story-specific questionnaires) and a scale of 1-7 (for reading habits) to match with the results from fMRI group. Due to data collection errors, ratings of "emotional" and "sad" from the rater group were excluded (marked by "*"). Unpaired t-tests assuming unequal variance were used, and final p-values were Bonferroni corrected by timing the total number of t-tests (48).

Supplementary Note: English translations of the literary stories

De Mexicaanse Hond (The Mexican Dog)

[Rating segment 1]

Mr. Kuisters from the fish shop, where **I/he** occasionally had to pick up sliced salmon for **my/his** mom on Friday afternoons after school, was a tall, bony man whose face consisted mainly of wrinkles. His head with its sheer ginger quiff was jutting out over his hunched shoulders. He cut the salmon with a thin knife that was worn in the middle. He took the slices between his thumb and index finger and carefully placed them side by side. His hands were purplish red and fish scales clung to them. He always asked **me/him** if **I/he** wanted to play with Tonia. **I/He** said **I/he** could not, because they were sitting at home waiting for the salmon.

[Rating segment 2]

But one day **I/he** could not escape. It was busy in the shop. As soon as Mr. Kuisters saw **me/him** come in he opened the sliding door between the shop and the apartment a bit and said that **I/he** should go inside because it would take quite a while. "Tonia is home." He pushed **my/his** shoulder and closed the door behind **me/him**. The room was dark and small. Tonia sat at the table staring at her hands lying in front of her on the plush tablecloth; a pale child with light, watery eyes and hair like flax. She was in **my/his** class and because everyone thought she not only resembled a fish, but also reeked of fish, no one wanted to play with her.

[Rating segment 3]

"What are you doing?" she asked. She put her hands in her lap and looked at **me/him** suspiciously. Her mouth was half open; her white face gleamed like it was smeared with grease.

"I have to wait here for your father."/He said that he has to wait for her father.

The furniture in the room was placed so close together that you could hardly walk without touching something. **I/He** shoved the chair on which **I/he** leaned **my/his** forearms as far as possible under the table, but when **I/he** leaned back a bit **I/he** felt the key of the dresser in **my/his** back. The space was further cramped by a huge tasseled lamp that hung like a parasol over **our/their** heads. On the edge of the chimney stood a black metal pendulum clock with a little naked man on top of the pendulum. He held a kind of club in one hand and with the other he pointed down to the dial. The clock ticked loudly. And **we/they** didn't say a word to each other. About twenty minutes later her father came in.

[Rating segment 4]

"Well," he said. He locked the door with a hook and changed his white coat for a brown jacket that hung on a hanger in the closet. "Now we can go peacefully about our business." He pushed a couple of chairs aside, which was the only way to get to the small table in the corner of the room. On top of the table stood an apparatus with a front plate made of black ebonite. Coils emerged out of it and it had two buttons at the bottom with a white scale. Mr. Kuisters pressed down a lever on the side of the device, turned the knobs, put the coils in a particular position, and asked his daughter if there was a new fuse in the control box.

[Rating segment 5]

She nodded.

"Wonderful," he said, "then we can start." He looked at his watch. "It is just the right time. Come over here." He beckoned to **me/him**, pulled a chair closer to the table and motioned to **me/him** that **I/he** should sit there. "Move your head slightly forward." He stood behind **me/him** and pushed gently against **my/his** crown, his hands went through **my/his** hair. A shiver crept down **my/his** spine up to **my/his** bottom. **I/He** smelled a sharp fishy smell that made **me/him** sick. The man put a double metal strap over **my/his** head and pressed two black discs with holes in them over **my/his** ears.

[Rating segment 6]

"Now listen," he cried, "here it comes." He leaned forward so that his big, saggy face hung in front of **mine/him**. I/He saw the red veins in his watery eyes and how his pupils darted back and forth - he wanted to see what **I/he** was hearing. An immense noise filled **my/his** ears, cracking, wheezing, tearing screams, long whistles, all of which suddenly merged into a furious roar that echoed through **my/his** whole body. Mr. Kuisters laughed. Now, that is him," he shouted, "that's the Mexican dog."

I/He tried to pull the discs from **my/his** ears, but he held them tight. He pressed his hand firmly on **my/his** head and turned one of the buttons. "Here's HDO, the Hilversum Radio Broadcasting," someone shouted, and after a few unintelligible phrases some deafening music hit against **my/his** eardrums, as if **my/his** head was jammed in the horn of **my/his** father's gramophone.

[Rating segment 7]

After a while, Mr. Kuisters abruptly let go of the headphones. He didn't seem pleased with the outcome. Dazed, **I/he** remained seated in the chair.

"Move." Tonia pulled **my/his** arm. "It is my turn now."

With ringing ears and throbbing temples **I/he** got up and walked to the door. With some difficulty, **I/he** loosened the hook. The door rumbled back on its tracks. The fishy smell in the shop was stronger than ever. **I/He** only dared to breathe again once I was outside, and **I/he** was already half a block away before **I/he** noticed that **I/he** had forgotten the salmon.

One afternoon **I/he** came home from school, and while **I/he** hung **my/his** coat on the rack in the hallway, **I/he** heard someone talking very loudly. The sound came from the living room. There were no other voices talking. It remained a grim monologue. Presuming that there was a visitor with a very unfriendly manner of conversation, or one of **my/his** relatives revealing the truth about what they think of **my/his** family, I/**he** gently opened the door and peeked inside.

[Rating segment 8]

Inside the room, **my/his** father and brother were standing on either side of the chimney. They had their heads slightly tilted and stared silently at the radio's speakers.

"Who is shouting?" **I/he** asked.

"That's Hitler," **my/his** father said. He gave **me/him** a sign to be quiet.

I/He remained there listening. It was only the first year that **I/he** had learned German at school, and **I/he** understood very little of it. **I/He** only understood the word "Juden", which the man uttered more and more often, in an increasingly contemptuous tone, as if he was kicking it. Even upstairs in **my/his** room **I/he** could hear his voice. His voice penetrated into every corner of the house. It even drowned out the sound of the rumbling sink faucet that **I/he** had opened to see whether **I/he** could still hear it against the running water.

[Rating segment 9]

I/He arranged **my/his** books and notebooks, but before **I/he** started my homework, **I/he** climbed up the attic stairs. **I/He** closed the attic door behind **me/him**. Without switching on the light **I/he** walked to the middle of the room and stood still. The sound of the voice was quieter here, but still very audible, and **I/he** went back to **my/his** room and started doing **my/his** homework. With **my/his** hands over **my/his** ears, **I/he** was trying to study **my/his** history lesson about the Holy Alliance. **I/He** felt the same sensation as years before at Mr. Kuisters' when **I/he** had the hard discs of the headset over **my/his** ears and heard the sound of radio for the very first time. The Mexican dog. **I/He** pressed **my/his** hands firmly against **my/his** ears, as if **I/he** subconsciously felt what that voice would bring.

De Muur (The Wall)

[Rating segment 1]

With **my/her** index finger **I/she** carefully **scratch/es** the leftover from the can. **My/her** ears register familiar sounds. The fizzing of the water in the toilets. The children's shrieks from the apartment above **my/her** head. The outside door three floors down that locks into place.

My/her old woman hands scrabble above the sink. The empty can falls on the ground and rolls with a bang against the metal trash can. My/her arm movement freezes. The man of the apartment next door must have heard the bang. The agonizing scratching will start again. Oh God, will this ever come to an end?

I lift/she lifts up **my/her** skirt, **lower/s myself/herself** to **my/her** hands and knees and **drag/s my/her** old body to the living room. **I/she need/s** to go there. Today, **I/she** will not let **myself/herself** be overtaken by fear.

For several days, the man from the apartment next door scratches on the wall. The scratching is destined for **me/her**. First and foremost, the man wants to frighten **me/her**. He then wants to break down the door. But this is **my/her** apartment. **I/she** won't let anybody in.

Every time the man hears **me/her** in the living room, the scratching starts. 'Do not make any more noise', is the message.

[Rating segment 2]

The scratching on the wall becomes more violent. The man acts as if he were a predator. He knows he can just scratch that wall without restraint, for **I/she** cannot ask a living soul for help.

I/she have/has to lure that man away from the wall. Now is the time to do so. Without any sound **I/she creep/s** towards the front door and **press/es my/her** eye against the round peephole. The elevator in the hallway produces a zooming sound. A woman gets out of the elevator and disappears into het apartment. A few minutes passed when **I/she** inaudibly **open/s** the door. **I/she** carefully **shuffle/s** across the hallway towards the next door. A single press on the bell. **I/she** then **run/s** back and **lock/s** the door.

The scratching stopped. The wall keeps silent. His doorbell must have caused some confusion. **I/she** did it. The trick is to distract a predator from its prey, you need to attract his attention to another part of his auditory field. A predator can only focus his hearing on one point only, it is his only weakness.

[Rating segment 3]

Don't make any more mistakes, do not direct his attention to the wall, be quiet as a mouse. Breathe out in six counts. Without a sound **I/she shift/s my/her** body weight backwards, only thereafter **I/she move/s**. This is the secret of cats.

Until recently **I/she** lived in the house **I/she** was born on the other side of the city. Sixty years **I/she** waited there for a man. While **I/she** waited, **I/she** read the announcements in the parish magazine and

watched the lottery draw on television. **I/she** read the horoscopes in the women's magazines. But no man ever appeared.

After the death of **my/her** parents **I/she** sold the house. With the money **I/she** bought this comfortable three room apartment and a Siamese cat. "The perfect companion", according to the shop assistant.

On hands and feet **I/she creep/s** to the kitchen, **grab/s** the pillcase and **fill/s** a glass of water. In the living room, **I/she crawl/s** upon the pillow. For a while **I/she lie/s** there motionless. **I/she** then **lick/s** the sleeping pill from her opened hand.

[Rating segment 4]

* * *

The scratching just woke **me/her** up. It's two o' clock. Never before had **I/she** heard the scratching noise during the night. **My/her** throat tightens. Rolled up **I/she lie/lies** on the pillow. **My/her** arms come into motion.

Tonight it will happen, this is the night the man has been waiting for. The neighbours have withdrawn themselves into the cocoon of sleep. **I/she** is all by **my/herself**. It's now solely between the man and **me/her**.

Away from the pillow, **(she)** mustn't remain lying on **my/her** back. He who lies on his back surrenders, even cats know that. **I/she** slowly **rise(s).**

Three minutes past two. Don't move. Count the minutes until the day breaks.

Three o' clock.

The scratching proceeded into clawing. What should **I/she** do? Cramps in **my/her** legs. Are there neighbours awake yet, or do they still wander in the soundless world of sleep. From which hour onwards will night no longer be night?

[Rating segment 5]

Four o'clock.

Cramped right leg. **I/she try/tries** to rely on **my/her** left leg. Not a single sound on the street. One more hour before the day breaks. Why did the scratching stop? Perhaps the man waited until fatigue numed **me/her**. Stay awake. He will not come in here.

Five o'clock.

The night is now really over. The neighbours awaken. They will hear **me/her** if **I/she cry/cries** for help. The man will take a risk if he chooses to invade **my/her** apartment at this time. Why does the morning

light not penetrate through the curtains yet, what is the morning light waiting for? Most crimes are committed at five o' clock, so **I/she** read in the newspaper.

Six o' clock.

A truck horns in the street, the city comes back to life. The neighbours come out of their bed. Why **do/does I/she** not hear anything? It's morning. It must be morning.

[Rating segment 6]

Seven o'clock.

The elevator comes into motion with a zooming sound. Somewhere above **my/her** head a child starts crying.

"Shut up!' someone shouts.

At last, the trusted sounds of the building. **I/she** survived the silence of the night. **I/she** drop/s my/herself to the pillow again, **pull/s my/her** legs up and **close/s my/her** eyes. The chains of sleep shan't disturb **me/her** no more.

Slowly **I/she open/s my/her** eyes. Sunbeams burn through the thin curtains. Someone flushes the toilet. A woman cries, a man laughs loud. The usual midday sounds. **I/she hoist/s** up **my/her body**, **stumble/s** to the kitchen and **open/s** a can.

[Rating segment 7]

The elevator in the hallway produces a zooming sound. Then it stops. Don't take any risk. **I/she have/has** to know what's going on there. Carefully creep towards the door. Voices in the hallway. **My/her** right eye peeks through the small round peephole. Two neighbours. 'Look, she's standing there again; says one woman, 'you can see the shadow of her feet under the door.'

My/her gaze slides to the bare strip between the bottom of the door and the doorstep.

'She's standing there quite frequently, says the other woman, often when I take the elevator.'

'That woman frightens me', says the first woman. 'I sometimes take the stairs because I am afraid to walk to the elevator'.

[Rating segment 8]

'It's a scary woman', says the first woman, 'she only leaves that apartment to go out for cat food. She talks to nobody.'

'She lives there all by herself with her Siamese cat', says the first voice.

Starteled **I/she move/s** away from the door. **My/her** sharp fingernails scratch **my/her** knees. On hands and feet **I/she** silently **creep/s** to the living room. By now **I /she know/s** how to do that.

I/she lay/s on the pillow and **roll/s my/herself** up. Why did this animal not want to share this pillow with **me/her**? Why did **I/she have/had** to throw out the cat just to keep this sleeping area in **my/her** own apartment?

Supplementary Figure 7: Correlations among behavioural measures in fMRI group

Spearman's rho heatmap

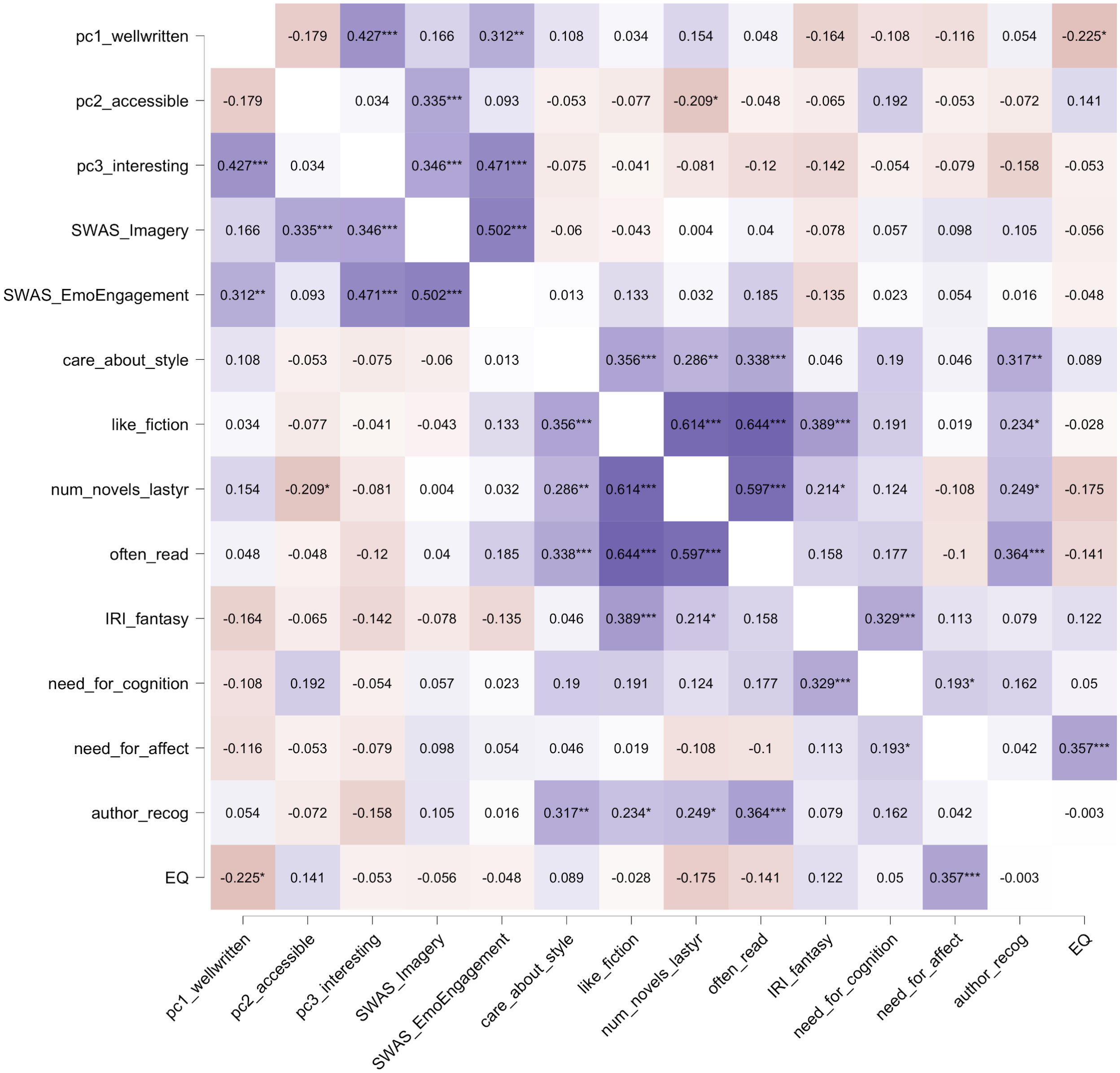

Supplementary Figure 7: Correlation matrix of story-specific and individual difference behavioural measures with one another.

Supplementary References

1. Forman, S. D. *et al.* Improved assessment of significant activation in functional magnetic resonance imaging (fMRI): use of a cluster-size threshold. *Magn. Reson. Med.* **33**, 636–647 (1995).
2. Slotnick, S. D. Cluster success: fMRI inferences for spatial extent have acceptable false-positive rates. *Cogn. Neurosci.* **8**, 150–155 (2017).
3. Lehne, M. *et al.* Reading a Suspenseful Literary Text Activates Brain Areas Related to Social Cognition and Predictive Inference. *PLOS ONE* **10**, e0124550 (2015).
4. Altmann, U., Bohrn, I. C., Lubrich, O., Menninghaus, W. & Jacobs, A. M. The power of emotional valence—from cognitive to affective processes in reading. *Front. Hum. Neurosci.* **6**, (2012).
5. Hagoort, P. MUC (Memory, Unification, Control) and beyond. *Front. Psychol.* **4**, (2013).
6. Wegrzyn, M., Herbert, C., Ethofer, T., Flaisch, T. & Kissler, J. Auditory attention enhances processing of positive and negative words in inferior and superior prefrontal cortex. *Cortex* **96**, 31–45 (2017).
7. Jenkins, A. C., Dodell-Feder, D., Saxe, R. & Knobe, J. The Neural Bases of Directed and Spontaneous Mental State Attributions to Group Agents. *PLOS ONE* **9**, e105341 (2014).
8. Kestemont, J. *et al.* Neural correlates of attributing causes to the self, another person and the situation. *Soc. Cogn. Affect. Neurosci.* **10**, 114–121 (2015).
9. Mason, R. A. & Just, M. A. The Role of the Theory-of-Mind Cortical Network in the Comprehension of Narratives. *Lang. Linguist. Compass* **3**, 157–174 (2009).
10. Xu, J., Kemeny, S., Park, G., Frattali, C. & Braun, A. Language in context: emergent features of word, sentence, and narrative comprehension. *NeuroImage* **25**, 1002–1015 (2005).

11. Bzdok, D. *et al.* Parsing the neural correlates of moral cognition: ALE meta-analysis on morality, theory of mind, and empathy. *Brain Struct. Funct.* **217**, 783–796 (2012).

12. Frith, C. d., Wolpert, D. m., Frith, U. & Frith, C. D. Development and neurophysiology of mentalizing. *Philos. Trans. R. Soc. Lond. B. Biol. Sci.* **358**, 459–473 (2003).

13. Mar, R. A. The Neural Bases of Social Cognition and Story Comprehension. *Annu. Rev. Psychol.* **62**, 103–134 (2011).

14. Saxe, R. & Kanwisher, N. People thinking about thinking peopleThe role of the temporo-parietal junction in “theory of mind”. *NeuroImage* **19**, 1835–1842 (2003).

15. Richardson, H. & Saxe, R. Development of predictive responses in theory of mind brain regions. *Dev. Sci.* **23**, e12863 (2020).

16. Mar, R. A., Oatley, K., Djikic, M. & Mullin, J. Emotion and narrative fiction: Interactive influences before, during, and after reading. *Cogn. Emot.* **25**, 818–833 (2011).

17. Hartung, F., Hagoort, P. & Willems, R. M. Readers select a comprehension mode independent of pronoun: Evidence from fMRI during narrative comprehension. *Brain Lang.* **170**, 29–38 (2017).

18. Wallentin, M. *et al.* Amygdala and heart rate variability responses from listening to emotionally intense parts of a story. *NeuroImage* **58**, 963–973 (2011).

19. Risius, U.-M. *et al.* Retrieval, Monitoring, and Control Processes: A 7 Tesla fMRI Approach to Memory Accuracy. *Front. Behav. Neurosci.* **7**, (2013).

20. St Jacques, P. L., Kragel, P. A. & Rubin, D. C. Dynamic neural networks supporting memory retrieval. *NeuroImage* **57**, 608–616 (2011).
