# Supplementary material for: Aesthetic appraisals of literary style and emotional intensity in narrative engagement are neurally dissociable
Source: Commun Biol. 2021 Dec 16;4:1401. doi: 10.1038/s42003-021-02926-0 (PMC8677754; doi:10.1038/s42003-021-02926-0)
Supplement: Supplementary file 3 — Description of Additional Supplementary Files [file 42003_2021_2926_MOESM3_ESM.pdf]

Description of Additional Supplementary Files

File name: Supplementary Data 1.

Description: Mean word ratings and levels of agreement (in Dutch).

File name: Supplementary Data 2.

Description: Spearman's Correlations among behavioural measures in fMRI group.
